# Technology-Based Interventions for Cancer Caregivers: Concept Analysis

**DOI:** 10.2196/22140

**Published:** 2021-11-16

**Authors:** Zhaohui Su, Xiaoshan Li, Dean McDonnell, Andrea A Fernandez, Bertha E Flores, Jing Wang

**Affiliations:** 1 Center on Smart and Connected Health Technologies School of Nursing University of Texas Health Science Center at San Antonio San Antonio, TX United States; 2 Program of Public Relations and Advertising Beijing Normal University-Hong Kong Baptist University United International College Zhuhai China; 3 Department of Humanities Institute of Technology Carlow Ireland; 4 School of Nursing University of Texas Health Science Center at San Antonio San Antonio, TX United States; 5 Florida State University College of Nursing Tallahassee, FL United States

**Keywords:** concept analysis, caregivers, cancer, oncology, technology-based interventions, mobile phone

## Abstract

**Background:**

Cancer is a taxing chronic disease that demands substantial care, most of which is shouldered by informal caregivers. As a result, cancer caregivers often have to manage considerable challenges that could result in severe physical and psychological health consequences. Technology-based interventions have the potential to address many, if not all, of the obstacles caregivers encounter while caring for patients with cancer. However, although the application of technology-based interventions is on the rise, the term is seldom defined in research or practice. Considering that the lack of conceptual clarity of the term could compromise the effectiveness of technology-based interventions for cancer caregivers, timely research is needed to bridge this gap.

**Objective:**

This study aims to clarify the meaning of technology-based interventions in the context of cancer caregiving and provide a definition that can be used by cancer caregivers, patients, clinicians, and researchers to facilitate evidence-based research and practice.

**Methods:**

The 8-step concept analysis method by Walker and Avant was used to analyze the concept of technology-based interventions in the context of cancer caregiving. PubMed, PsycINFO, CINAHL, and Scopus were searched for studies that examined technology-based interventions for cancer caregivers.

**Results:**

The defining attributes of technology-based interventions were recognized as being accessible, affordable, convenient, and user-friendly. On the basis of insights gained on the defining attributes, antecedents to, and consequences of technology-based interventions through the concept analysis process, technology-based interventions were defined as the use of technology to design, develop, and deliver health promotion contents and strategies aimed at inducing or improving positive physical or psychological health outcomes in cancer caregivers.

**Conclusions:**

This study clarified the meaning of technology-based interventions in the context of cancer caregiving and provided a clear definition that can be used by caregivers, patients, clinicians, and researchers to facilitate evidence-based oncology practice. A clear conceptualization of technology-based interventions lays foundations for better intervention design and research outcomes, which in turn have the potential to help health care professionals address the needs and preferences of cancer caregivers more cost-effectively.

## Introduction

### Background

Cancer does not discriminate—it is prevalent across demographics and geographies [1]. Cancer is also pernicious—it could overwhelm the physiological health and psychological well-being of patients with cancer and cancer caregivers [2-7]. Informal caregivers, for instance, often have to shoulder a considerable amount of care burden—depending on the disease trajectory of the patients, approximately 55%-95% of caregivers shoulder mental health disorders such as distress [8-10]. In the context of this study, the term *health care professionals* describes health care personnel, including doctors, nurses, and all other formal caregivers, whereas *informal cancer caregivers*, *cancer caregivers*, and *caregivers* are used interchangeably, referring to informal cancer caregivers such as family and friends, who often regularly provide a wide range of assistance to a patient with cancer. Although, overall, a variety of interventions hold promise to alleviate caregiver burden, ranging from print materials and face-to-face consultations to telephone-based assistance [11-20], technology-based interventions are considered the most practical and promising solution available to caregivers.

### The Critical Role of Technology-Based Interventions

The emphasis on technology-based interventions for cancer caregivers has become particularly pronounced amid the COVID-19 pandemic, a global health crisis that has effectively crippled many, if not all, of the traditional health care services available to patients and caregivers [21-23]. During the pandemic, many cancer caregivers have found much-needed solace and support in technology-based health care services, ranging from online support groups to videoconferencing with patients or health care professionals [24-26]. It is important to note that there is a growing body of research investigating the benefits of technology-based health solutions [24-29]. For instance, a systematic review revealed that caregivers significantly improved their cancer knowledge and communication outcomes after receiving technology-based interventions [27]. Throughout the pandemic, many scholars worried about whether the lack of *personal touch* might undermine technology-based interventions [28]. However, it is worth noting that, although face-to-face interactions have advantages, the social dynamics of these consultations could also hinder health care outcomes. For instance, in a study that compared the intervention efficacy of face-to-face consultations and technology-based interventions, researchers found that, among these 2 types of interventions, caregivers were more likely to truthfully report their stress symptoms to a web-based support system and have these symptoms addressed and treated [29].

### The Importance of Conceptual Clarity

Although research on technology-based interventions for caregivers is gaining momentum, it faces many obstacles [30]. One of the most prominent hurdles that could considerably undermine the research field is the lack of a clear and consistent definition of the term *technology-based interventions*. It is important to note that, although the application of technology-based interventions is on the rise, the term is seldom defined when applied in cancer research or practice. A review of the literature [31-34] shows that alarmingly, much of the research on technology-based interventions for patients with cancer fails to provide a clear definition of the term to shed light on key questions: (1) Are technology-based interventions the same as terms such as *web-based interventions*? (2) What are the key characteristics of technology-based interventions? (3) What constitutes a technology-based intervention? The lack of conceptual clarity of the term *technology-based interventions* could substantially undermine the research field, as one of the most espoused truisms in academia is arguably that, particularly in light of scientific integrity and solidarity, scholars cannot measure what they cannot define [35-37]. As one scholar, the prominent British physicist and mathematician Lord William Thomson Kelvin, succinctly put it, *“*What is not defined cannot be measured. What is not measured cannot be improved. What is not improved is always degraded*”* [38].

### Technology-Based Interventions and Related Terms

#### Overview

Before further elaborating on the urgent need for a clear definition of the term *technology-based interventions*, it is critical to shed light on why there is an urgent need to analyze and define the concept—similar terms (eg, digital health) applied in the research field often harbor deep-rooted issues that could cause confusion among scholars. Overall, a kaleidoscope of terms, such as *digital health*, *eHealth*, and *mobile health (mHealth*), has been used to describe a wide range of health solutions available to cancer caregivers [39-43]. These terms often refer to health solutions in the form of health services or products that are enabled by the internet (eg, emails and web-based appointments), multifunctional devices that are elevated by the connectivity of the internet (eg, smartphones such as the iPhone), or tools and services built upon other networking opportunities (eg, Amazon devices, such as Echo and Tile, developed on low-bandwidth networks such as the Sidewalk framework [44] or Bluetooth technologies). On the surface, these terms seem to describe various technology-based interventions in accordance with their unique characteristics, such as how the term mHealth can be used to refer to smartphone-based health interventions. However, a closer examination of these concepts reveals deep-rooted research issues.

#### Too Broad, Too Narrow, and Too Many Overlaps in Related Terms

To begin with, because of a lack of clear and consistent definitions, these terms can mean different things to different audiences—depending on the specific research contexts, they can be either extremely broad or narrow given that their meanings could vary widely as the research contexts shift (eg, example applications [39-43]). This is particularly true as technology-based tools or services become increasingly flexible and versatile. For instance, depending on the research context, terms such as *digital health*, *eHealth*, and *mHealth* can refer to a broad spectrum of health solutions, ranging from video-based materials on self-care or cancer care management (eg, television programs), web- or telephone-based communication with a wider support circle (eg, health care professionals), journaling in any or many enabling devices, or a hybrid or multicomponent intervention that consists of divergent forms of technology-based interventions [39-43].

At the same time, these terms can be too narrow. For instance, *mHealth* is often adopted to describe smartphone-, tablet-, and app-based health solutions [41] but not for interventions that involve laptop computers or smartwatches, even though they both possess similar defining functions to those of smartphones and tablets (eg, devices that can be easily carried and work on the go). The same applies for terms such as *digital health*, *eHealth*, *mHealth*—as researchers or caregivers’ definitions of *digital* vary, for instance, *digital health* can refer to network connectivity in one study and to characteristics of the intervention or the delivery platform in another [45-48]. These *too broad* or *too narrow* issues lead to the conclusion that these terms might be further complicated by the fact that these terms are often not mutually exclusive [32,49,50]. For instance, video-based interventions can be delivered via DVD, television, computer, smartphone, or even electronic health records [49], which means that, because of a lack of conceptual clarity, these interventions can be described as any of the following: digital health, eHealth, or mHealth interventions. Overall, in contrast to technology-based interventions, terms such as *digital health*, *eHealth*, and *mHealth* are plagued by (1) a lack of definition and consensus regarding the scope of *digital health*, *eHealth,* and *mHealth*; (2) the absence of consistency in the interpretations of the meanings of *digital*, *electronic*, and *mobile*; and (3) the flexibility and versatility of technology opportunities that are often categorized as *digital health*, *eHealth*, and *mHealth* (eg, video-based interventions that can be delivered via mobile devices, desktop computers, and televisions).

It is important to underscore that these drawbacks also apply to terms such as *technology-mediated interventions*, *internet-based interventions*, and *web-based interventions* that have been used in cancer research [51], in contrast to more embracing terms such as *technology-based* and concepts such as *mediated*, *web*, or *internet* that are more flexible, versatile, and open to interpretation. Overall, compared with terms such as *digital*, *electronic*, and *mobile*, *technology* is a more focused and confined description of health solutions that incorporate technological elements. In other words, even though it also lacks conceptual clarity, the term *technology-based interventions* only faces one issue: the lack of a clearly defined conceptualization. These insights combined underscore the importance of establishing conceptual clarity for the term *technology-based interventions first,* before venturing into research on broader concepts such as *digital health*, *eHealth*, and *mHealth*.

### Technology-Based Interventions: The Need for Conceptual Clarity

One of the most concerning phenomena in cancer research on technology-based interventions is the fact that several studies have investigated the concept without clearly defining and delineating its conceptual parameters [52-55]. In other words, without a clearly delineated conceptual definition of the term, a wide range of measurements have been used for technology-based interventions [30]. This practice is extremely worrisome and problematic. Without large-scale systematic reviews or meta-analysis studies [56-58], it is difficult to determine the degree of discrepancies between the true effects of technology-based interventions and what has been measured and reported. What is clear, however, is that the lack of definitions, compounded by the heterogeneity of the measures adopted to gauge the barely or poorly defined concept, could substantially undermine the reproducibility and replicability of research on technology-based interventions [56-58], not to mention the quality of review studies on technology-based interventions for cancer caregivers.

The importance of reproducibility and replicability in research cannot be overstated [35]. These 2 research criteria are indispensable to scientific research, ranging from concept building, evidence collection, and data analysis to the interpretation and application of research findings [35-37]. In essence, reproducibility and replicability are instrumental in advancing the literature, elevating the research field, and building the collective knowledge base of the society [35]. However, because of barely or poorly defined key research concepts, researchers might risk missing the valuable opportunity to (1) understand and interpret current research findings on technology-based interventions for cancer caregivers, (2) pinpoint effective components of the interventions, and (3) apply these components to future intervention studies to further the research field [35-37]. Thus, to bridge the research gap, this study aims to examine technology-based interventions in the context of cancer caregiving via the lens of concept analysis.

### Objective

The aim of our study is to explore the meaning of technology-based interventions in the context of cancer caregiving and provide a definition.

## Methods

### Concept Analysis

One of the most well-accepted and widely adopted approaches to establish conceptual clarity is concept analysis [59-61]. Concept analysis is an important analytical tool in understanding the nuanced conceptual and theoretical meaning of a term [59], which could be understood as a research process that “entails the systematic examination of the attributes or characteristics of a given concept for the purpose of clarifying the meaning of that concept” [61]. Conceptual clarity of key research variables is indispensable to the development of science and research. In other words, concept analysis generates a structured meaning that establishes rules and guidelines for the correct use and applications of the concept. In this study, the concept analysis method was adopted to clarify the meaning of technology-based interventions in the context of cancer caregiving and to provide a definition that can be used by cancer caregivers, patients, clinicians, and researchers to facilitate evidence-based research and practice.

### Technology-Based Interventions

**Figure 1:**
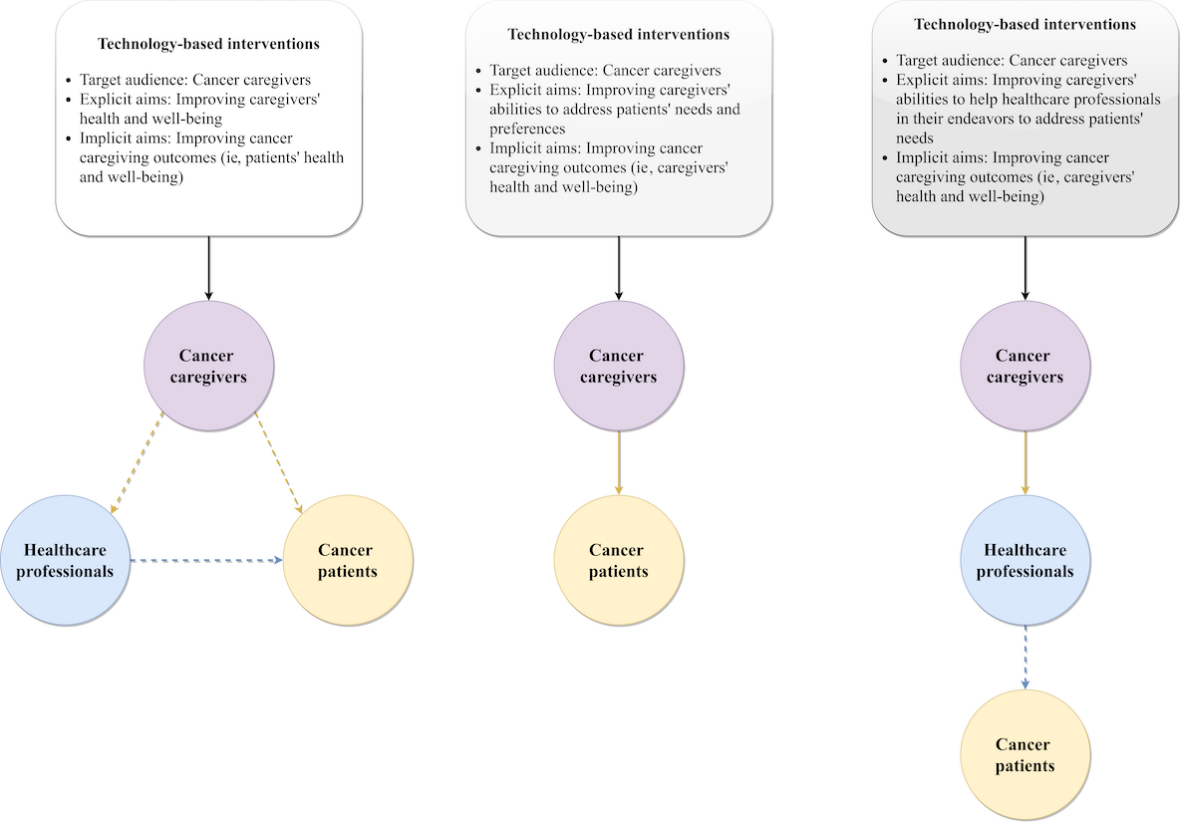
A framework of subgroups of technology-based interventions with cancer caregivers as the target audience.

A review of the literature shows that technology-based interventions for cancer caregivers can be categorized into 3 groups in terms of the explicit aims they focus on the following: (1) helping the caregivers themselves, (2) helping caregivers help the patients, and (3) helping caregivers to facilitate the abilities of health care professionals to improve the patient-provider relationship or the health outcomes of patients with cancer. On the surface, these 3 subgroups of technology-based interventions for cancer caregivers seem to have substantial divergences. However, it is important to note that the similarities between these subgroups are more pronounced and meaningful: (1) all of these interventions have cancer caregivers as their first-degree target audience, (2) these subgroups share the same intervention mechanisms, and (3) their overall aims are in line with one another—to improve the abilities of caregivers, patients, and health care professionals to better address the caregiving needs and preferences of patients with cancer and in turn, patients health and quality of life. Thus, all these subgroups of interventions were considered in this study. A framework that can help health care professionals better understand these interventions is shown in Figure 1.

### Theoretical Framework

Although there are many concept analysis approaches available in the literature, the method by Walker and Avant [59] was adopted as the theoretical framework in this study. The decision was based on the following considerations: (1) the method by Walker and Avant is the most used concept analysis framework [60]—and adopting this method could help facilitate research replicability in the field, (2) using a method that the audience is familiar with can help the readership better focus on the gist of the study—clarifying and defining the concept of technology-based interventions in cancer caregiving, which in turn could (3) help readers better understand the need for a clear definition of technology-based interventions and the merits of the concept analysis methodology, and (4) the method by Walker and Avant is more linear and structured compared with other models [62], which can help researchers build a more straightforward presentation of the research process and study findings.

**Figure 2:**
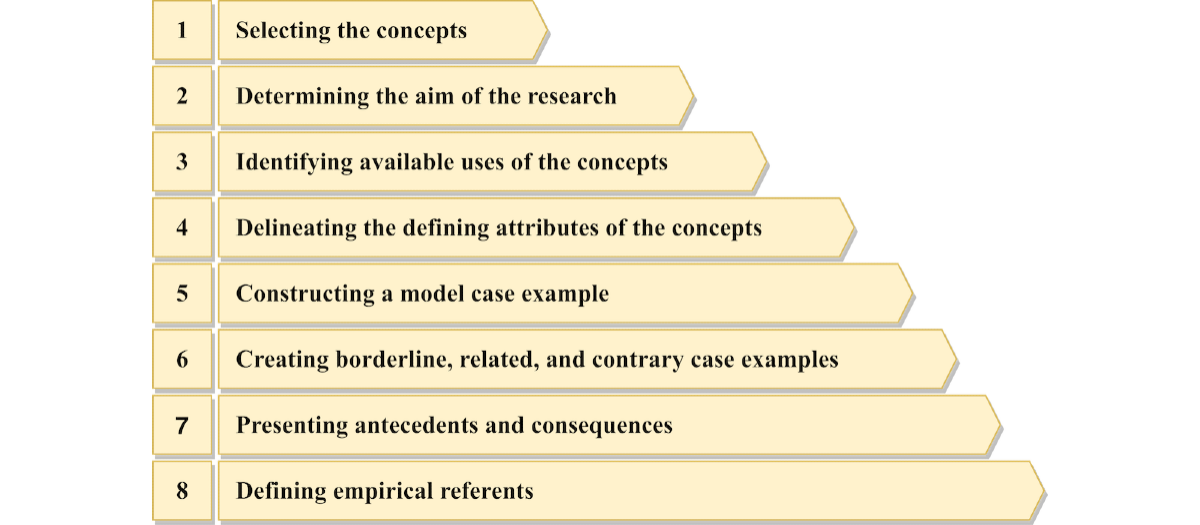
A schematic representation of the concept analysis procedures adopted in the study.

There are 8 steps in the concept analysis method by Walker and Avant [59]: (1) selecting the concepts; (2) determining the aim of the research; (3) identifying available uses of the concepts; (4) determining the defining attributes of the concepts; (5) constructing a model case example; (6) creating borderline, related, and contrary case examples; (7) presenting antecedents and consequences; and (8) defining empirical referents. The definitions of key concept analysis terms adopted in this study can be found in Textbox 1. To better illustrate the research procedures, we also created a schematic figure to delineate the methodological steps we took to obtain our research findings (Figure 2).

**Definitions of key terms of the concept analysis method adopted in the study.**

**Concept and definition**

- Defining attribute: recurring characteristics of the concept
- Antecedent: occurrence that happened before, and that directly shape, the concept
- Consequence: occurrence that happened as a result of, and are directly influenced by, the concept
- Model case: real-life and often paradigmatic use of concept cases that reflects the essence of the concept
- Related case: cases that have characteristics that are similar to the concept at face value but are different from the concept at its core upon close examination
- Borderline case: cases that contain most, but not all, of the key attributes of the concept
- Contrary case: cases that represent what the concept is not (eg, have little or none of the defining attributes of the concept)
- Empirical referent: real-world phenomena that demonstrate the concept

### Search Strategy and Data Analysis

On the basis of the guidelines by Walker and Avant [59], a literature synthesis was adopted to capture available conceptual dimensions of technology-based interventions. An extensive and cross-disciplinary review of the literature was conducted to capture the full breadth of technology-based interventions. Partially because of a lack of relevant literature, publications in the fields of computer science, psychology, and behavioral sciences were all included in the review. The databases PubMed, PsycINFO, CINAHL, and Scopus were searched between June and July 2020. The search terms used were as follows: *(cancer/tumor) AND (caregiver/carer/family/spouse/partner) AND (technology-based intervention OR trial/treatment/therapy)*; search terms varied slightly in different databases.

Both the research objectives and search terms were developed in 2 stages. The first research stage was where we accidentally encountered the conceptualization issue associated with the term *technology-based interventions*. Our initial research objective was to conduct a systematic review study on technology-based interventions for cancer caregivers [24]. During this process, we found that, although there is a rich body of research on technology-based interventions for cancer caregivers, most of the authors fail to offer a clear conceptualization of the term. As we delved deeper into the issue, we realized that our team also had yet to develop a clearly delineated definition of technology-based interventions—we assumed that we knew what we ventured out to study. This revelation, combined with insights gained from additional research on the subject matter, yielded the conclusion that a concept analysis study was needed to proceed with our original research plan, which was contingent on an evidence-based and clearly defined conceptualization of the term *technology-based interventions.* Thus, to address this research gap, we conducted this study. To date, 3 sets of search terms have been developed and used specifically for this study: 1 for the systematic review, 1 to search for definitions, and 1 for our concept analysis.

**Figure 3:**
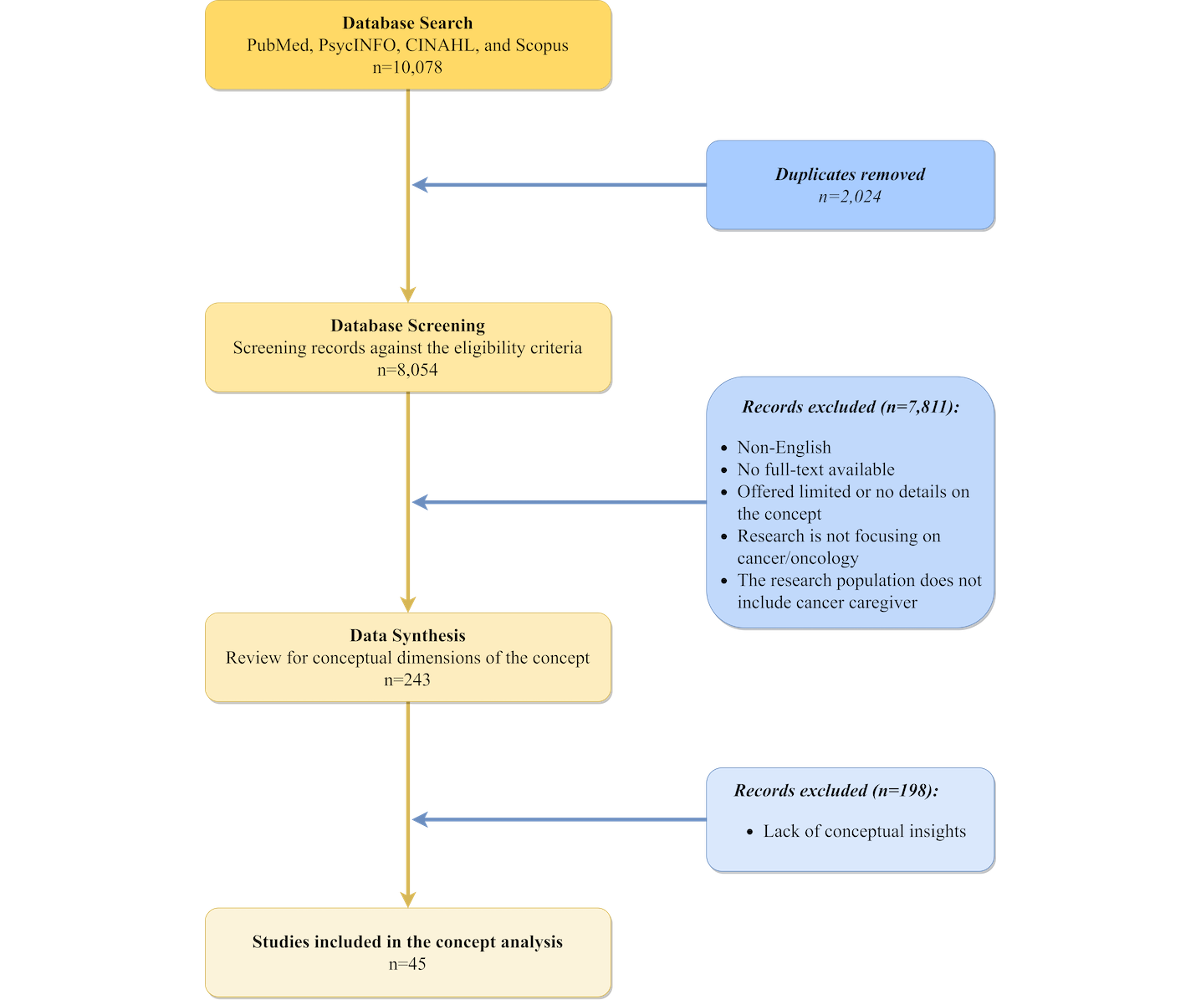
Data screening and analysis flowchart.

The search terms were developed based on insights gained from the literature, web-based group discussions, and brainstorming sessions (including all authors and the school’s academic librarian) as well as examples set by previous literature [63]. Articles were reviewed for broad research focus (eg, research context and design) and detailed descriptions of technology-based interventions (eg, the role of technology in the intervention). Key information (eg, use of technology and intervention content) from eligible articles was extracted and analyzed. Two principal reviewers (ZS and XL) conducted the review. Discrepancies were resolved via group discussions that included all authors until a consensus was reached. Through this process of synthesis and comparison, a clear conceptualization of the term emerged. The details of the data screening and analysis processes are illustrated in Figure 3.

### Eligibility Criteria

Articles were excluded if they failed to provide conceptual insights on technology-based interventions; more specifically, the exclusion criteria were as follows: the study was (1) not written in English, (2) not peer-reviewed, (3) not focusing on technology-based interventions (eg, papers focusing on face-to-face strategies for cancer caregivers), and (4) not centering on cancer caregivers. The inclusion criteria are listed in Textbox 2.

**Study inclusion criteria.**

**Inclusion criteria**

- Participants: informal cancer caregivers
- Language: English
- Study type: journal articles
- Study context: discussing technology-based interventions for cancer caregivers
- Intervention: technology-based; cancer caregivers being either the sole or one of the key target audiences

## Results

### Overview

The reviewed articles consisted of titles, abstracts, and full-text articles in English from 2010 to 2020, resulting in 10,078 records. The key articles included in the review are listed in Multimedia Appendix 1 [19,27,31-34,45,64-101]. A total of 45 articles met the eligibility criteria (Multimedia Appendix 1). In addition, a manual search of the reference lists of eligible articles located further articles of relevance. Drawing insights from the literature [102-104], Google Scholar was used to reverse-trace articles that cited papers included in the final review as an additional measure to ensure a comprehensive literature search strategy. On the basis of the study results, the concept of technology-based interventions was defined as the use of technology to design, develop, and deliver health promotion contents and strategies aimed at inducing or improving positive physical or psychological health outcomes in cancer caregivers. In the following sections, detailed information on the use of the concept, defining attributes, relevant cases, antecedents and consequences, as well as empirical referents is presented and discussed.

### Use of the Concept

Overall, the available definitions of technology-based interventions often revolve around 2 components: the use of technology and the purpose of the intervention. Limited emphasis placed on aspects such as the integration of technology into the intervention or end-user involvement in the application of the technology complicates the research area. When examining the effectiveness of behavioral interventions, researchers define technology-based interventions as approaches that use “information and communication technology applications to promote behavioral outcomes” [105]. Researchers also discussed technology-based interventions in terms of the technology platforms they adopted. In a study focusing on mental health, the term *technology-based intervention* is used synonymously with the concept of *internet-based interventions* [106]. The study outlines that both approaches *include computer-based and web-based interventions, text messaging, interactive voice recognition, smartphone apps, and emerging technologies* [48].

Some definitions allow technology platforms integrated with technology-based interventions to be more inclusive, where platforms such as computers, web-based apps, mobile phones, and wearable sensors are all considered possible venues for intervention delivery [107-110]. In addition to the emphasis on the use of technology, technology-based interventions are often defined with a focus on intervention objectives and projected outcomes. Aiming to examine the influence of an intervention on informal caregivers of stroke survivors, researchers describe technology-based interventions as “some form of telepractice that uses information and communication technologies to help eliminate distance barriers and to help with scheduling logistics, thus extending the scope for provision of quality healthcare” [111]. Overall, although promising studies are emerging in the literature, there is a dearth of insights that could provide conceptual clarity to the term *technology-based interventions*, particularly in the cancer caregiving research field.

### Defining Attributes

Defining attributes are recurring themes that mirror *the heart of concept analysis* [43]. On the basis of insights gained from the literature review and data synthesis, *accessible* [64,65,112], a*ffordable* [66,112], *convenient* [66,67,113], and *user-friendly* [40,68-71,114] were identified as the defining attributes of technology-based interventions. Although additional characteristics were identified, these attributes were the most frequent traits found across the interventions analyzed. One of the key attributes of technology-based interventions was accessibility: compared with conventional solutions, technology-based interventions can be accessed whenever and wherever [64,65,112]. In other words, cancer caregivers can access technology-based interventions without having to worry about transportation or other logistical issues (eg, availability of appointments).

The second defining attribute of technology-based interventions was affordability. In addition to resources related to transportation, considering that many technology-based interventions can be accessed free of cost (eg, smartphone app [115]), caregivers often do not have to worry about financial resources needed for them to adopt these interventions [66,112]. The ability to be accessed whenever and wherever and often without charge subsequently makes technology-based interventions convenient to use and access [66,67,113]. In addition to these traits (ie, accessible, affordable, and convenient), technology-based interventions often adopt a user-friendly design to improve user engagement [40,68-71,114], such as incorporating gamification mechanisms that can improve the user experience of cancer caregivers while learning ways to improve their health and well-being.

Another aspect of being user-friendly centered on the respect technology-based interventions have for end-user input—some interventions were developed in a co-design fashion, where health promotion strategies were discussed and built by cancer caregivers, health care professionals, and academic scholars collaboratively [69]. This method is an important participatory approach for intervention development, and it has many advantages, the most noticeable ones centering on the ability of the co-design to yield more optimal anticipated outcomes and less unintended consequences compared with interventions that only involve limited groups of stakeholders [116-118]. Although it is difficult to determine which of these defining attributes is the most appealing to cancer caregivers, it is clear that these characteristics have collectively made technology-based interventions appealing to cancer caregivers.

### Relevant Cases

#### Model Case and Contrary Case

To make the comparison more apparent, an example scenario that incorporates these 2 types of cases is constructed in this paper. The cases were developed according to the instructions given by Walker and Avant [59] and insights were drawn from the literature [119-121]. The first example relates to usual care and is the contrary case. At the same time, resources such as *Doctor Carer*, which possess the key defining attributes of technology-based interventions by being accessible, affordable, convenient, and user-friendly, are the example of the model case. Details of the example case can be found in Textbox 3.

**Details of the model and contrary case example.**

**Case example**

Angie is a 35-year-old Latina living in a rural Texas city that has a well-built Hispanic community. She has been worrying ever since she was informed that her mother has cervical cancer. After her brother died in a factory accident, Angie became the breadwinner of her family; she works 3 jobs to support her parents and her 2 adolescent children from a previous marriage. Though self-reliant, Angie often feels helpless, as she knows nothing about how to take care of her mother or how to establish a functioning *new normal* for her family. Angie wishes she lived outside of a rural context; traveling 200 miles to and from the closest cancer clinic has a taxing impact on her family and her career. Help and hope seem to be too far away. Angie shared her concerns with a woman she met at the clinic. Eva, now her best friend, showed Angie free resources available via smartphone. Angie was overwhelmed. Using her smartphone, Angie registered with almost all available cancer websites, watched hundreds of hours of YouTube tutorials and caregiver stories, and downloaded over 2 dozen medical apps on her phone to learn more about how to be a caregiver to her mother. Angie just downloaded an app called *Doctor Carer*, which can connect her with volunteer cancer doctors for free. She hopes this app can provide her with the answers she desperately needs and bring her one step closer to feeling less overwhelmed.

#### Borderline Case and Related Case

According to Walker and Avant [59], a borderline case could be understood as a case with most but not all defining attributes of the concept. In contrast, a related case has traits that are similar but different from those inherent to the concept. The aim of developing the following scenario, one that embodies both a borderline case and a related case, is to compare and contrast these 2 types of cases. In contrast to the cases mentioned in the section *Model Case and Contrary Case*, the comparison in this section will focus on the influence of the caregiver on the patient. In this scenario, the borderline case is represented by the communication between Kacey (the patient with cancer) and her friend Ann (the cancer caregiver), whereas the related case is depicted by Ann’s use and adoption of the interactive multimedia e-book, *Compendium of Materia Medica*. Details of the borderline case and related case examples are presented in Textbox 4. To further shed light on these 4 types of cases and their connected functionality in explaining the concept of technology-based interventions, a comparison of the model case, contrary case, related case, and borderline case was conducted and is discussed in Table 1.

**Details of the example borderline and related cases.**

**Case example**

Kacey is a 25-year-old aspiring actress living in Los Angeles, California. She is also a patient with breast cancer; diagnosed with stage I breast cancer a week ago. Although the diagnosis brought chaos to Kacey’s life, her social support systems have kept her afloat. Ann, Kacey’s best friend since high school, has been an unwavering source of support to Kacey. Whenever Kacey is in distress, Ann is there for her, talking, videoconferencing, and interacting on social media with her to help her weather through tough times. Kacey is unable to afford insurance and, therefore, is uninsured for the moment. Disappointed by the limited resources that are available to her, Kacey was determined to find alternative health care resources she could explore. Recently, she was mesmerized by the documentaries and books Ann shared with her. Kacey was impressed by what the documentaries argued, and she has planned to stop consuming meat and adopt a vegan diet starting next week. She intends to use the rest of this week to design her own diet. Kacey bought one of the e-books Ann mentioned to her, *Compendium of Materia Medica*, as soon as she read its description. The book has a very detailed account of foods that have beneficial properties to the human body, along with suggestions on what to eat under various circumstances. The book is better than an encyclopedia; it has texts, illustrations, and interactive media embedded in it to enhance the learning experience. Kacey knows she has a long fight ahead of her. But she is hopeful.

**Table 1:** Comparison of the differences among the model case, contrary case, related case, and borderline case.

Parameter	Model case	Contrary case	Related case	Borderline case
Definition	Real-life and often paradigmatic use of concept cases that reflects the essence of the concept	Case that represent what the concept is not—have little or none of the defining attributes of the concept.	Case that have characteristics similar to the concept at face value but different from the concept at its core upon close examination.	Case that contain most, but not all, of the key attributes of the concept.
Example	Resources like *Doctor Carer* mentioned in Angie’s caregiving experience	Usual care mentioned in Angie’s caregiving experience.	Ann’s use and adoption of the interactive multimedia e-book *Compendium of Materia Medica.*	The communication between Ann and her friend Kacey.
Defining attribute	The use of technology to design, develop, and deliver health promotion contents and strategies aimed at inducing or improving positive physical or psychological health outcomes in cancer caregivers	In-person communicated and delivered health promotion contents and strategies; no technology is involved.	Nontailored interventions that are not designed, developed, or delivered based on Ann’s needs and preferences as Kacey’s informal cancer caregiver	Not all caregiver–patient communication is about the caregiving experience or the cancer continuum, enabled or delivered via technology.
Detailed rationale	*Doctor Carer* is an intervention that possesses all the defining attributes of technology-based interventions.	No technology is needed for in-person communicated interventions to occur, which means that, although it is an intervention nonetheless, it is not a technology-based intervention.	Like all interventions, technology-based interventions are intentionally designed and delivered to address the needs and wants of caregivers. Either the book *Compendium of Materia Medica* or its digitalization is intentionally created with caregivers like Ann in mind.	For Ann, communicating with Kacey can occur either in person or via technology-based methods, and it may not necessarily have an impact on her caregiving experience.

### Antecedents and Consequences

In this section, whenever antecedents and consequences are mentioned, they refer to *antecedents to technology-based interventions* and *consequences of technology-based interventions*, respectively. Two types of antecedents to technology-based interventions were identified. First, antecedents to the need for interventions involve factors such as cancer-related psychosocial distress [72] and lack of couple-based interventions [65]. Second, antecedents to the adoption of technology-based interventions operate as opposed to conventional interventions and take into consideration the physical or geographical constraints [64] and the prevalence of technology, such as smartphones [66]. In addition, resonating with these antecedents, 2 types of consequences of technology-based interventions were found. First, consequences of the intervention stimuli as a whole addressed aspects such as improved quality of life [68] and reduced stress [69] among caregivers. Second, focused on consequences of the use of technology-based interventions rather than conventional interventions such as positive Google Analytics results [69] and intention to use the telemedicine tool in the future [67]. Detailed information on the identified antecedents and consequences is presented in Table 2.

**Table 2:** Antecedents to and consequences of technology-based interventions.

Type		Category
**Antecedents**		
	Antecedents to the need for interventions	Cancer-related psychosocial needs [72] Lack of couple-based interventions [65] Neglect of psychosocial concerns of family caregivers [87]
	Antecedents to the need for technology-based interventions	Physical constraints [64] Prevalence of smartphones [66] Feasibility of internet- or web-based interventions [71]
**Consequences**		
	Consequences of the intervention as a whole	Improved quality of life [68] Reduced stress [91] Improved marital communication, confidence, and skills [85]
	Consequences of the use of technology-based interventions	Positive Google Analytics results [69] Intention to use the app in the future [67] Bring positive effect or healthier psychosocial states in patients [76]

### Empirical Referents

Empirical referents can be considered real-world demonstrations of a concept [59]. For technology-based interventions, empirical referents can be interventional medical apps developed for caregivers. In 2017, there were an estimated 325,000 medical apps available on smartphones, which could translate into over 3.7 billion medical app downloads among smartphone users [122]. Of these 325,000 apps, those that are commercially available, interventional in nature, and designed for cancer caregivers could be considered empirical referents to technology-based interventions.

## Discussion

### Principal Findings

Although technology-based interventions are essential to health care research and practice, there is a lack of definition of the concept, particularly in the context of cancer caregiving. In this paper, we set out to clarify the meaning of technology-based interventions in the context of cancer caregiving and provide a definition that can facilitate evidence-based oncology research and practice. Considering that the lack of conceptual clarity of the term could undermine the effectiveness of technology-based interventions in addressing the health challenges of cancer caregivers, timely research is needed to bridge the gap. To the best of our knowledge, this is the first study to examine technology-based interventions from a concept analysis perspective. Aiming to obtain conceptual clarity for the term, we adopted the method by Walker and Avant [59] as the guiding framework; carefully reviewed the literature; identified defining attributes; and developed key case examples, antecedents, and consequences that are indispensable to the conceptual infrastructure of technology-based interventions.

**Figure 4:**
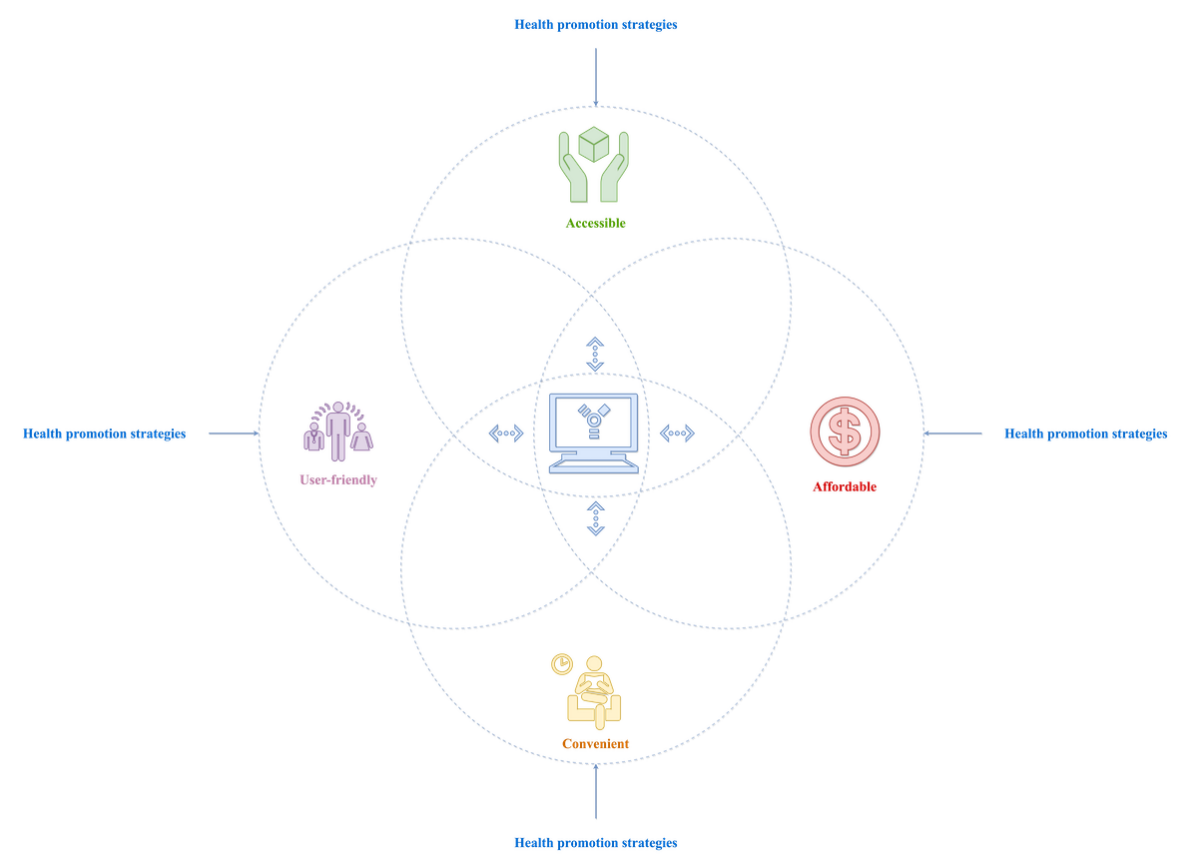
A schematic representation of the technology-based intervention attributes.

The key defining attributes that characterize technology-based interventions are *accessible*, *affordable*, *convenient*, and *user-friendly*. Combining the identified antecedents and consequences, the following definition was proposed: technology-based interventions are defined as the use of technology to design, develop, and deliver health promotion contents and strategies aimed at inducing or improving positive physical or psychological health outcomes in cancer caregivers. A detailed illustration of the interplay of the key defining attributes that characterize the concept of technology-based interventions is shown in Figure 4. Overall, Figure 4 underscores that, in essence, technology-based interventions are health promotion strategies augmented with technology platforms to make them more effective (ie, accessible, affordable, convenient, and user-friendly) in improving the health and well-being of cancer caregivers.

This definition and the defining attributes could be a solution to address some of the critical issues regarding the conceptualization of the term, both in the current and broader research contexts of technology-based interventions [123,124], which compromises the ability of the existing research to enrich the literature. A growing number of papers have begun to acknowledge and address the importance of adopting clear and structured methodological procedures and frameworks to ensure research reproducibility and replicability [125,126]. The absence of a clear definition could lead to poor replicability and low comparability of intervention studies, which in turn, limits the applicability and generalizability of these studies and their corresponding interventions [35]. Viewed as a mechanism to connect current research findings and generate new insights, systematic review research has the potential to further contribute to the growth of research inquiry [56].

However, evidence suggests that systematic review studies often fall victim to the lack of conceptual definitions in the literature [126]. Results show that 40%-89% of poorly described interventions are not replicable, which means that they cannot be adequately used in systematic reviews or offer substantial contributions to the development of the research field [127]. The availability of a clear definition of the research topic enables research studies to report their findings accurately and meaningfully to facilitate further research endeavors, such as systematic reviews and meta-analysis studies [126,127]. From this perspective, the results of this study offer opportunities to address key methodological issues in the literature, such as a lack of conceptual definitions of technology-based interventions in cancer caregiving research. By offering a clear and concise definition of technology-based interventions that clarifies the process using systematically identified antecedents, defining attributes, and consequences, the findings of this study can help guide future interventions that aim to improve the well-being and health outcomes of cancer caregivers.

The findings of this study underscore that technology-based interventions should be clearly conceptualized in terms of the following aspects: (1) the use of technology in the intervention (ie, as the communication platform), (2) the key components the intervention incorporates (ie, technology as the communication platform and health promotion strategies as the content), (3) the relationship between the key components (ie, a communication platform and content symbiosis; the role of technology is flexible, ranging from *managing* to *supporting* the intervention content), (4) the purpose of the intervention (ie, to produce health solutions for cancer caregivers), and (5) the defining characteristics of technology-based interventions (ie, accessible, affordable, convenient, and user-friendly key traits inherent to technology and the audience-centered communication approach). Overall, the insights provided by this study can help researchers better understand and interpret outcomes and technology-based interventions, identify effective intervention strategies, and apply them to future studies that have the potential to further improve the health outcomes of cancer caregivers.

### Limitations

Although this study fills significant voids in the literature, it is not without limitations. A concept analysis approach was adopted in this study to conduct a structured and comprehensive literature search. We conducted our literature search in the PubMed, PsycINFO, CINAHL, and Scopus databases for eligible articles and manually screened the articles that were referenced or cited in these articles. Although these databases are comprehensive, it is possible that articles were indexed exclusively in other databases that were not included in the analysis. We did not follow the PRISMA (Preferred Reporting Items for Systematic Reviews and Meta-Analyses) procedures [128] in presenting our data screening process. Rather, we modeled our flowchart based on example concept analyses [129] that used a more linear and simplified data screening process. Although our choice of data screening flowchart was justified, we understand that this screening procedure may not meet the expectations of some readers. In our future research endeavors, we will adopt the PRISMA procedures to ensure detailed screening information is presented in the manuscript. Finally, this concept analysis only included articles published in English. This eligibility criterion may further limit our data pool.

### Conclusions

Technology-based interventions play an increasingly important role in addressing the health and well-being of caregivers across the cancer continuum. Although technology-based interventions can offer substantial benefits to patients with cancer and their caregivers, many limitations could hinder the design, development, and deployment of these interventions. The results of our study offer much-needed conceptual clarity on the term, which in turn, could help build a more rigorous and robust research environment for investigations on technology-based interventions, both in the context of cancer caregiving and beyond. Overall, conveying a clear definition of technology-based interventions to researchers, health care practitioners, and cancer caregivers is a foundational step in establishing a collaborative and coordinated effort to develop and deploy cost-effective interventions. On the basis of the study findings, technology-based interventions are defined as the use of technology to design, develop, and deliver health promotion contents and strategies aimed at inducing or improving positive physical or psychological health outcomes in cancer caregivers. We believe this definition serves as a key step toward a mutual ground that elevates comparability between interventions and outcomes, which in turn, could further advance the research field and the knowledge base.

## Appendix

Multimedia Appendix 1 Key articles included in the review.

## Abbreviations

mHealth: mobile health
