# Supplementary material for: Technology-Based Interventions for Cancer Caregivers: Concept Analysis
Source: JMIR Cancer. 2021 Nov 16;7(4):e22140. doi: 10.2196/22140 (PMC8663574; doi:10.2196/22140)
Supplement: Multimedia Appendix 1 [file cancer_v7i4e22140_app1.docx]

**Multimedia Appendix 1.** Key articles included in the review

| **Author** | **Year** | **Title** |
| --- | --- | --- |
| Ali et al. [1] | 2019 | User acceptance of an app-based adherence intervention: Perspectives from patients taking oral anticancer medications |
| Applebaum et al. [2] | 2018 | Exploring the cancer caregiver’s journey through web-based meaning-centered  psychotherapy |
| Atreya et al. [3] | 2019 | Being present: A single-arm feasibility study of audio-based mindfulness meditation for colorectal cancer patients and caregivers |
| Badr et al. [4] | 2016 | Development and usability testing of a web-based self-management intervention for oral cancer survivors and their family caregivers |
| Carr et al. [5] | 2019 | Impact of a mobilized stress management program (Pep-Pal) for caregivers of oncology patients: Mixed-methods study |
| Carrion-Plaza et al. [6] | 2020 | HabitApp: Newplay technologies in pediatric cancer to improve the psychosocial state of patients and caregivers |
| Dalal et al. [7] | 2016 | A web-based, patient-centered toolkit to engage patients and caregivers in the acute care setting: A preliminary evaluation |
| Demiris et al. [8] | 2018 | Innovative tools to support family caregivers of persons with cancer: The role of information technology |
| Dionne-Odom et al. [9] | 2016 | Family caregiver depressive symptom and grief outcomes from the ENABLE III randomized controlled trial |
| DuBenske et al. [10] | 2010 | Web-based cancer communication and decision making systems: Connecting patients, caregivers, and clinicians for improved health outcomes |
| Duggleby et al. [11] | 2017 | Feasibility study of an online intervention to support male spouses of women with breast cancer |
| Dykes et al. [12] | 2014 | Participatory design and development of a patient-centered toolkit to engage hospitalized patients and care partners in their plan of care |
| Gramatges et al. [13] | 2018 | Improving childhood cancer survivor care through web-based platforms |
| Heynsbergh et al. [14] | 2018 | Feasibility, useability and acceptability of technology-based interventions for informal cancer carers: A systematic review |
| Kaltenbaugh et al. [15] | 2015 | Using web-based interventions to support caregivers of patients with cancer: A systematic review |
| Köhle et al. [16] | 2018 | Intent to use a web-based psychological intervention for partners of cancer patients: Associated factors and preferences |
| Kubo et al. [17] | 2018 | A pilot mobile-based mindfulness intervention for cancer patients and their informal caregivers |
| Kubo et al. [18] | 2019 | A randomized controlled trial of mHealth mindfulness intervention for cancer patients and informal cancer caregivers: A feasibility study within an integrated health care delivery system. |
| Kurahashi et al. [19] | 2018 | The perceived ease of use and usefulness of loop: evaluation and content analysis of a web-based clinical collaboration system |
| Lapid et al. [20] | 2015 | Cancer caregiver: Perceived benefits of technology |
| Lewis et al. [21] | 2017 | Taking Care of Her: A pilot feasibility study of a caregiver intervention for women with advanced stage ovarian cancer |
| Marzorati et al. [22] | 2018 | Telemedicine use among caregivers of cancer patients: Systematic review |
| Northouse et al. [23] | 2014 | A tailored web-based psychoeducational intervention for cancer patients and their family caregivers |
| Ownsworth et al. [24] | 2020 | Use of telehealth platforms for delivering supportive care to adults with primary brain tumors and their family caregivers: A systematic review |
| Park et al. [25] | 2014 | Effects of my child's safety web-based program for caregivers of children with cancer in South Korea |
| Pensak et al. [26] | 2017 | Development of a web-based intervention for addressing distress in caregivers of patients receiving stem cell transplants: Formative evaluation with qualitative interviews and focus groups |
| Reblin et al. [27] | 2018 | Feasibility of implementing an electronic social support and resource visualization tool for caregivers in a neuro-oncology clinic |
| Santin et al. [28] | 2019 | The development of a web-based resource to provide information and psychosocial support to informal cancer carers in hospitals in Vietnam |
| Santin et al. [29] | 2020 | Using a six-step co-design model to develop and test a peer-led web-based resource (PLWR) to support informal carers of cancer patients |
| Silveira et al. [30] | 2011 | Cancer Carepartners: Improving patients' symptom management by engaging informal caregivers |
| Shen et al. [31] | 2017 | Telehealth in older adults with cancer in the United States: The emerging use of wearable sensors |
| Shin et al. [32] | 2018 | Supporting caregivers of patients with cancer: A summary of technology-mediated interventions and future directions |
| Song et al. [33] | 2015 | Improving couples' quality of life through a web-based prostate cancer education intervention |
| St. George et al. [34] | 2020 | Development of a multigenerational digital lifestyle intervention for women cancer survivors and their families |
| Steel et al. [35] | 2016 | Web-based collaborative care intervention to manage cancer-related symptoms in the palliative care setting |
| Sterba et al. [36] | 2019 | Evaluation of a survivorship needs assessment planning tool for head and neck cancer survivor-caregiver dyads |
| Sun et al. [37] | 2019 | Improving family caregiver and patient outcomes in lung cancer surgery: Study protocol for a randomized trial of the multimedia self-management (MSM) intervention |
| Tang et al. [38] | 2014 | Web-based interventions for caregivers of cancer patients: A review of literatures |
| Voruganti et al. [39] | 2017 | My Team of Care study: A pilot randomized controlled trial of a web-based communication tool for collaborative care in patients with advanced cancer |
| Walsh et al. [40] | 2014 | Home medication support for childhood cancer: Family-centered design and testing |
| Wang et al. [41] | 2015 | Developing "Care Assistant": A smartphone application to support caregivers of children with acute lymphoblastic leukaemia |
| Wang et al. [42]. | 2018 | mHealth supportive care intervention for parents of children with acute lymphoblastic leukemia: Quasi-experimental pre- and postdesign study |
| Washington et al. [43] | 2018 | Delivering problem-solving therapy to family caregivers of people with cancer: A feasibility study in outpatient palliative care |
| Wolpin et al. [44] | 2015 | Development and usability testing of a web-based cancer symptom and quality-of-life support intervention |
| Zulman et al. [45] | 2012 | Adapting an in-person patient-caregiver communication intervention to a tailored web-based format |

**References:**

1. Ali, E.E., et al., *User acceptance of an app-based adherence intervention: Perspectives from patients taking oral anticancer medications.* J Oncol Pharm Pract, 2019. **25**(2): p. 390-397.

2. Applebaum, A.J., et al., *Exploring the cancer caregiver's journey through web-based Meaning-Centered Psychotherapy.* Psychooncology, 2018. **27**(3): p. 847-856.

3. Atreya, C.E., et al., *Being present: A single-arm feasibility study of audio-based mindfulness meditation for colorectal cancer patients and caregivers.* PLoS ONE, 2018.

4. Badr, H., et al., *Development and usability testing of a web-based self-management intervention for oral cancer survivors and their family caregivers.* Eur J Cancer Care (Engl), 2016. **25**(5): p. 806-21.

5. Carr, A.L., et al., *Impact of a mobilized stress management program (Pep-Pal) for caregivers of oncology patients: Mixed-methods study.* JMIR Cancer, 2019. **5**(1): p. e11406.

6. Carrion-Plaza, A., J. Jaen, and I. Montoya-Castilla, *HabitApp: Newplay technologies in pediatric cancer to improve the psychosocial state of patients and caregivers.* Frontiers in Psychology, 2020. **11**(157).

7. Dalal, A.K., et al., *A web-based, patient-centered toolkit to engage patients and caregivers in the acute care setting: A preliminary evaluation.* J Am Med Inform Assoc, 2016. **23**(1): p. 80-7.

8. Demiris, G., et al., *Innovative tools to support family caregivers of persons with cancer: The role of information technology.* Seminars in Oncology Nursing, 2019. **35**(4): p. 384-388.

9. Dionne-Odom, J.N., et al., *Family caregiver depressive symptom and grief outcomes from the ENABLE III randomized controlled trial.* Journal of Pain and Symptom Management, 2016. **52**: p. 378-385.

10. DuBenske, L.L., et al., *Web-based cancer communication and decision making systems: Connecting patients, caregivers, and clinicians for improved health outcomes.* Med Decis Making, 2010. **30**(6): p. 732-44.

11. Duggleby, W., et al., *Feasibility study of an online intervention to support male spouses of women with breast cancer.* Oncol Nurs Forum, 2017. **44**(6): p. 765-775.

12. Dykes, P.C., et al., *Participatory design and development of a patient-centered toolkit to engage hospitalized patients and care partners in their plan of care.* AMIA Annu Symp Proc, 2014. **2014**: p. 486-95.

13. Gramatges, M.M., et al., *Improving childhood cancer survivor care through web-based platforms.* Oncology (Williston Park), 2018. **32**(1): p. e1-e10.

14. Heynsbergh, N., et al., *Feasibility, useability and acceptability of technology-based interventions for informal cancer carers: A systematic review.* BMC Cancer, 2018. **18**(1): p. 244.

15. Kaltenbaugh, D.J., et al., *Using Web-based interventions to support caregivers of patients with cancer: A systematic review.* Oncol Nurs Forum, 2015. **42**(2): p. 156-64.

16. Köhle, N., et al., *Intent to use a web-based psychological intervention for partners of cancer patients: Associated factors and preferences.* J Psychosoc Oncol, 2018. **36**(2): p. 203-221.

17. Kubo, A., et al., *A pilot mobile-based mindfulness intervention for cancer patients and their informal caregivers.* Mindfulness, 2018.

18. Kubo, A., et al., *A randomized controlled trial of mHealth mindfulness intervention for cancer patients and informal cancer caregivers: A feasibility study within an integrated health care delivery system.* Integrative Cancer Therapies, 2019.

19. Kurahashi, A.M., et al., *The perceived ease of use and usefulness of loop: evaluation and content analysis of a web-based clinical collaboration system.* JMIR Hum Factors, 2018. **5**(1): p. e2.

20. Lapid, M.I., et al., *Cancer caregiver: Perceived benefits of technology.* Telemed J E Health, 2015. **21**(11): p. 893-902.

21. Lewis, F., et al., *Taking Care of Her: A pilot feasibility study of a caregiver intervention for women with advanced stage ovarian cancer.* Journal of Cancer Therapy, 2017. **8**: p. 472-489.

22. Marzorati, C., et al., *Telemedicine use among caregivers of cancer patients: Systematic review.* J Med Internet Res, 2018. **20**(6): p. e223.

23. Northouse, L., et al., *A tailored Web-based psychoeducational intervention for cancer patients and their family caregivers.* Cancer Nurs, 2014. **37**(5): p. 321-30.

24. Ownsworth, T., et al., *Use of telehealth platforms for delivering supportive care to adults with primary brain tumors and their family caregivers: A systematic review.* Psychooncology, 2021. **30**(1): p. 16-26.

25. Park, B.K. and E. Lee, *Effects of my child's safety web-based program for caregivers of children with cancer in South Korea.* Healthc Inform Res, 2014. **20**(3): p. 199-208.

26. Pensak, N.A., et al., *Development of a web-based intervention for addressing distress in caregivers of patients receiving stem cell transplants: Formative evaluation with qualitative interviews and focus groups.* JMIR Res Protoc, 2017. **6**(6): p. e120.

27. Reblin, M., et al., *Feasibility of implementing an electronic social support and resource visualization tool for caregivers in a neuro-oncology clinic.* Support Care Cancer, 2018. **26**(12): p. 4199-4206.

28. Santin, O., et al., *Using a six-step co-design model to develop and test a peer-led web-based resource (PLWR) to support informal carers of cancer patients.* Psycho-Oncology, 2019. **28**(3): p. 518-524.

29. Santin, O., et al., *The development of a web-based resource to provide information and psychosocial support to informal cancer carers in hospitals in Vietnam.* Psycho-Oncology, 2020. **n/a**(n/a).

30. Silveira, M.J., et al., *Cancer Carepartners: Improving patients' symptom management by engaging informal caregivers.* BMC Palliat Care, 2011. **10**: p. 21.

31. Shen, J. and A. Naeim, *Telehealth in older adults with cancer in the United States: The emerging use of wearable sensors.* J Geriatr Oncol, 2017. **8**(6): p. 437-442.

32. Shin, J.Y., et al., *Supporting caregivers of patients with cancer: A summary of technology-mediated interventions and future directions.* Am Soc Clin Oncol Educ Book, 2018. **38**: p. 838-849.

33. Song, L., et al., *Improving couples' quality of life through a web-based prostate cancer education intervention.* Oncology Nursing Forum, 2015.

34. St. George, S.M., et al., *Development of a multigenerational digital lifestyle intervention for women cancer survivors and their families.* Psycho-Oncology, 2020. **29**(1): p. 182-194.

35. Steel, J.L., et al., *Web-based collaborative care intervention to manage cancer-related symptoms in the palliative care setting.* Cancer, 2016. **122**(8): p. 1270-82.

36. Sterba, K.R., et al., *Evaluation of a survivorship needs assessment planning tool for head and neck cancer survivor-caregiver dyads.* J Cancer Surviv, 2019. **13**(1): p. 117-129.

37. Sun, V., et al., *Improving family caregiver and patient outcomes in lung cancer surgery: Study protocol for a randomized trial of the multimedia self-management (MSM) intervention.* Contemp Clin Trials, 2019. **83**: p. 88-96.

38. Tang, W.P., et al., *Web-based interventions for caregivers of cancer patients: A review of literatures.* Asia Pac J Oncol Nurs, 2014. **1**(1): p. 9-15.

39. Voruganti, T., et al., *My Team of Care study: A pilot randomized controlled trial of a web-based communication tool for collaborative care in patients with advanced cancer.* J Med Internet Res, 2017. **19**(7): p. e219.

40. Walsh, K.E., et al., *Home medication support for childhood cancer: Family-centered design and testing.* J Oncol Pract, 2014. **10**(6): p. 373-6.

41. Wang, J., et al., *Developing "Care Assistant": A smartphone application to support caregivers of children with acute lymphoblastic leukaemia.* J Telemed Telecare, 2016. **22**(3): p. 163-71.

42. Wang, J., et al., *Mhealth supportive care intervention for parents of children with acute lymphoblastic leukemia: Quasi-experimental pre- and postdesign study.* JMIR mHealth and uHealth, 2018.

43. Washington, K.T., et al., *Delivering problem-solving therapy to family caregivers of people with cancer: A feasibility study in outpatient palliative care.* Psychooncology, 2018. **27**(10): p. 2494-2499.

44. Wolpin, S.E., et al., *Development and usability testing of a web-based cancer symptom and quality-of-life support intervention.* Health Informatics J, 2015. **21**(1): p. 10-23.

45. Zulman, D.M., et al., *Adapting an in-person patient-caregiver communication intervention to a tailored web-based format.* Psychooncology, 2012. **21**(3): p. 336-41.
